# Longitudinal trajectory characteristics of the erythropoietin resistance index and their association with all-cause mortality in maintenance hemodialysis patients

**DOI:** 10.1080/0886022X.2026.2756708

**Published:** 2026-11-10

**Authors:** Yun Yang, Wang He, Junbo Shi, Xiaoyun Wang, Feng Pan, Anrong Tong

**Affiliations:** ^a^ Department of Nephrology, Ningxia Hui Autonomous Region Hospital of Traditional Chinese Medicine (Ningxia Hui Autonomous Region Research Institute of Chinese Medicine), Yinchuan, China; ^b^ College of Traditional Chinese Medicine, Ningxia Medical University, Yinchuan, China

**Keywords:** Renal anemia, maintenance hemodialysis, erythropoietin resistance index, mortality

## Abstract

**Objective:**

Anemia is common in kidney failure, and most patients depend on erythropoiesis-stimulating agents (ESAs). We grouped maintenance hemodialysis (MHD) patients by how their erythropoietin resistance index (ERI) changed over time, and asked whether these patterns predicted death.

**Methods:**

We reviewed records of MHD patients treated at our center between 2021 and 2025. K-means clustering was applied to repeated ERI measurements to define trajectories. Logistic regression tested whether trajectory group, continuous ERI, and ERI quartile were related to all-cause mortality, the primary outcome. We also used spline models, subgroup and sensitivity analyses, and examined whether ERI improved prediction.

**Results:**

A total of 150 patients were included, and 110 had enough repeated ERI data for trajectory analysis; 26 of them died (23.6%). Baseline ERI quartiles differed in dialysis vintage, dry weight, hemoglobin, and sex. In adjusted models, none of the ERI measures—continuous, quartile, or trajectory—predicted mortality. Dialysis vintage, sex, and hemoglobin were stronger predictors, and adding ERI barely changed discrimination.

**Conclusions:**

ERI varied widely between patients and tracked the severity of anemia and overall clinical state. It did not, however, show an independent association with death that we could detect. With a retrospective design and limited precision, our results are inconclusive about prognostic value rather than evidence that none exists.

## 1. Introduction

Epidemiological studies in recent years have demonstrated that chronic kidney disease (CKD) is a public health problem of major international concern [1,2]. According to the Global Burden of Disease Study 2017, there are 132.3 million people with CKD in China, which is the highest in the world [3]. Maintenance hemodialysis (MHD) is one of the main renal replacement therapies (RRT) used in renal failure. Renal anemia is the most common complication among patients with CKD and those on MHD. Patients may suffer from dizziness and fatigue, which can affect their quality of life, and may also be a burden on the heart and raise the risk of death [4,5]. It is mainly related to low renal erythropoietin production, iron deficiency, low-grade inflammation and malnutrition.

The mainstay of treatment for renal anemia in MHD patients is erythropoiesis-stimulating agents (ESAs) [6,7]. Despite regular and adequate ESA therapy, however, some patients continue to have hemoglobin levels outside of the target range, which is clinically known as low responsiveness to ESAs or erythropoiesis resistance. Low ESA responsiveness is a sign of suboptimal treatment effect on anemia, also be a reflection of complex pathological conditions in patients, including iron metabolism abnormalities, increased inflammatory burden, poor nutritional status, or inadequate dialysis adequacy. The erythropoietin resistance index (ERI) is a common index to measure the ESA responsiveness, and has been widely applied in recent years to evaluate the ESA treatment response and clinical prognosis of MHD patients. It should be noted that ESA hyporesponsiveness is a clinical state defined by the failure to achieve target hemoglobin levels despite adequate ESA therapy, whereas the ERI is a quantitative index calculated as the weekly ESA dose normalized by post-dialysis body weight and hemoglobin level; the former is a categorical clinical judgment, while the latter grades the degree of responsiveness continuously and can therefore be tracked over time.

Numerous studies have used various methods to investigate the factors influencing ERI and its relationship with prognosis; however, several limitations remain. Most studies have used baseline ERI or static ERI as exposure variables, making it difficult to reflect the dynamic changes in ESA responsiveness among MHD patients during long-term dialysis. Previous studies have largely focused on the linear relationship between ERI and prognosis or on differences between high- and low-ERI groups based on cutoff points, with few efforts to further identify patient subgroups with different longitudinal ERI patterns; consequently, it has been difficult to elucidate the heterogeneous characteristics of ESA resistance in MHD patients. Furthermore, there remains a lack of systematic evaluation regarding whether ERI trajectory grouping can further improve the predictive ability for mortality risk beyond traditional clinical indicators. In addition, most previous reports came from single-center cohorts with modest sample sizes, and their reported positive associations between higher ERI and mortality may have been confounded by differences in follow-up duration, case mix, and ESA prescription patterns; reverse causation, in which a higher ERI reflects greater underlying illness severity rather than contributing to death, also cannot be excluded.

This study retrospectively enrolled clinical data from MHD patients in our dialysis unit from 2021 to 2025. We cleaned, merged, and organized the raw quarterly data to construct databases for quarterly, annual, and patient-level analyses. Based on early-year ERI levels, we used clustering methods to identify different longitudinal ERI trajectories among MHD patients and compare differences in baseline clinical characteristics across different trajectory groups. Building on this, the study further employed generalized linear and logistic regression models to analyze the association between ERI and mortality outcomes from the perspectives of ERI trajectory groups, continuous ERI levels, and ERI quartile groups, respectively. Through restricted cubic spline analysis, subgroup analysis, sensitivity analysis, and predictive gain analysis, the study systematically evaluated the predictive value of dynamic ERI characteristics for the clinical prognosis of MHD patients. Through this study, we aimed to provide new clinical evidence for the early identification of ESA resistance, individualized management of anemia, and risk stratification for mortality in MHD patients. The specific hypothesis of this study was that (1) distinct longitudinal ERI trajectories can be identified among MHD patients, and (2) patients with a persistently high ERI trajectory have higher all-cause mortality than those with a persistently low ERI trajectory.

## 2. Methods

### 2.1. Data sources

This study is a retrospective observational study conducted at the Dialysis Center of Ningxia Hui Autonomous Region Hospital of Traditional Chinese Medicine (Ningxia Hui Autonomous Region Research Institute of Chinese Medicine and). Patients who underwent maintenance hemodialysis at this center from 2021 to 2025 were included. The inclusion criteria were as follows: (1) age > 18 years, (2) hemodialysis treatment duration > 3 months with a stable clinical condition, (3) use of arteriovenous fistula, and (4) anuria. The exclusion criteria were as follows: (1) severe cardiovascular or cerebrovascular diseases, (2) infectious diseases within one month, (3) active liver diseases or cancer, (4) recent blood transfusion or surgical procedures, (5) active hemorrhage or pure red cell aplasia, (6) no treatment with rHuEPO, and (7) limb deficiency. After applying the above criteria, 150 patients were included in the final analysis. Of these, 110 patients with sufficient longitudinal ERI data were included in the trajectory analysis. During follow-up, 26 deaths occurred (19 in the low-ERI trajectory group and 7 in the high-ERI trajectory group).

All patients received hemodialysis 3 times per week for 4 h each time, using sugar-free bicarbonate dialysates and heparin anticoagulants during hemodialysis. The dialysate flow was 500 mL/min, and the blood flow was 200–350 mL/min. The dialysate ingredients were as follows: sodium 140 mmol/L, potassium 2.0 mmol/L, calcium 1.25 mmol/L, and magnesium 0.5 mmol/L. This study was approved by the Ethics Committee of the Ningxia Hui Autonomous Region Hospital of Traditional Chinese Medicine (Ningxia Hui Autonomous Region Research Institute of Chinese Medicine) (Approval No. 2026-143) and was carried out in accordance with the Declaration of Helsinki. Because the analysis was retrospective and used only de-identified data from routine dialysis records, the waiver involved no more than minimal risk to the patients, and it did not adversely affect their rights or welfare. No personally identifiable information is included in this manuscript. the committee of Ningxia Hui Autonomous Region Hospital of Traditional Chinese Medicine (Ningxia Hui Autonomous Region Research Institute of Chinese Medicine) waived the need for written informed consent.

### 2.2. Data collection

Clinical data for each of the four quarters of every year were collected, including demographic information such as age, sex, body weight, dialysis vintage, clinical outcomes, and rHuEPO dosage. All laboratory parameters were recorded, including pre-dialysis serum albumin, prealbumin, creatinine, blood urea nitrogen (BUN), alkaline phosphatase (ALP), cholesterol, triglycerides, serum potassium, serum sodium, carbon dioxide binding capacity, phosphorus, total calcium, high-sensitivity C-reactive protein (hs-CRP), intact parathyroid hormone (iPTH), ferritin, serum iron, transferrin saturation, spKt/V, and URR. A complete blood count (CBC) was also performed prior to hemodialysis. Pre-dialysis blood samples were collected from the venous system of dialysis patients. The raw Excel data were broken down by quarter, consolidated across multiple years, standardized for variable naming, processed for outliers, and processed for missing values to construct analysis databases at the raw visit level, patient quarterly level, patient annual level, and patient level. All patients were treated with recombinant human erythropoietin (rHuEPO), which was administered intravenously at the end of each hemodialysis session two to three times per week, with the dose individually titrated according to hemoglobin response. Hemoglobin and other laboratory parameters were measured in pre-dialysis blood samples collected at the beginning of each session, and therefore reflected the pre-dose baseline status rather than any immediate post-dose effect.

### 2.3. Variables

1. Calculation of ERI: The erythropoietin resistance index (ERI) was used to assess EPO responsiveness. ERI is a widely recognized and effective indicator for evaluating EPO responsiveness, defined as follows: ERI = weekly EPO dose (U/week)/post-dialysis body weight (kg)/Hb (g/L) [8]. Because hemoglobin is expressed in g/L throughout this study, the resulting ERI is numerically one tenth of the value obtained when hemoglobin is expressed in g/dL, which is the convention used in most previous reports; to convert the values reported here to that convention, multiply by 10 (for example, the baseline median of 2.55 in the lowest quartile corresponds to 25.5 U/kg/week per g/dL). Baseline ERI was defined as the first available ERI measurement at study entry, and the early mean ERI was defined as the average of all ERI measurements recorded during the early observation period (the first study year) for each patient.
2. Outcome Variables: All-cause mortality was the primary outcome during the follow-up period. Because of the absence of precise event onset times, logistic regression analysis was used as the main statistical tool to evaluate associations with the outcome.
3. Trajectory Grouping Method

Based on early ERI levels collected between 2021 and 2025, K-means clustering was used to identify patients’ longitudinal ERI patterns. The optimal number of clusters was determined by considering the sum of squares within clusters, the silhouette coefficient, the minimum cluster proportion, and clinical interpretability.

### 2.4. Statistical analysis

Continuous variables were expressed as mean ± standard deviation or median (interquartile range) based on their distribution characteristics; categorical variables were expressed as frequency (percentage). When comparing baseline characteristics between groups, an independent-samples t-test or analysis of variance (ANOVA) was used for normally distributed continuous variables; the Mann–Whitney U test or Kruskal–Wallis test was used for non-normally distributed continuous variables; and the chi-square test or Fisher’s exact test was used for categorical variables. Specifically, K-means clustering was applied to each patient’s standardized annual mean ERI values, with multiple random initializations to avoid local optima, and the number of clusters was prespecified to range from two to four to ensure a minimum cluster size adequate for between-group comparisons.

Trend charts were used to describe the quarterly and annual changes in ERI. Based on annual ERI data from 2021 to 2025, clustering methods were employed to identify longitudinal ERI change patterns among patients and to group them into trajectories accordingly. Further comparisons were made of baseline clinical characteristics and future mortality rates across different trajectory groups.

The associations between ERI trajectory groups, continuous ERI measures and ERI quartile groups with mortality outcomes were analyzed using multivariate logistic regression, adjusting for potential confounding factors, including age, sex, dialysis vintage, albumin, hemoglobin, and ferritin. In addition, restricted cubic spline analysis was used to investigate the nonlinear association between ERI and mortality outcomes.

Subgroup analyses and interaction tests use to assess the consistency of associations across different clinical characteristics; sensitivity analyze, log-transformed ERI, were conducted to test the robustness of the results. ROC curves, AUC, and DCA were used to evaluate the additional predictive value of ERI and its trajectory information relative to the baseline clinical model. All statistical tests were two-sided, and a P value < 0.05 was considered statistically significant. No formal *a priori* sample size or power calculation was performed because this was a retrospective, hypothesis-generating analysis; a total of 26 deaths occurred, and with seven parameters in the multivariable models the events-per-variable ratio was approximately 3.7, which is below the conventional threshold of 10, indicating limited statistical precision and a potential risk of overfitting. Because exact dates of death were not systematically recorded, logistic regression rather than time-to-event analysis was used as the primary analytical framework. All patients were observed over the same calendar period (2021–2025) to minimize follow-up imbalance; the potential bias arising from unequal individual follow-up durations is acknowledged in the Discussion.

## 3. Results

### 3.1. Comparison of baseline characteristics by baseline ERI quartile

After dividing the study participants into four groups (Q1–Q4) based on baseline ERI levels, significant differences were observed in some baseline characteristics among the groups (Table 1, Figures S1a–c). The number of cases in each quartile was generally similar, suggesting that the groups were well balanced (Figure S1a). Further comparison of baseline clinical characteristics revealed statistically significant differences in dialysis vintage across the different baseline ERI quartiles. The median dialysis vintage in the Q1, Q2, Q3, and Q4 groups was 43.00, 62.00, 56.50, and 61.00, respectively, suggesting that patients in higher ERI groups tended to have longer dialysis vintage (*p* = 0.009). At the same time, dry weight differed significantly across the different ERI quartiles, showing an overall downward trend, with the Q1 group having the highest dry weight and the Q4 group having the lowest (*p* < 0.001). Hemoglobin levels also gradually decreased as ERI quartiles increased; the median hemoglobin levels in the Q1, Q2, Q3, and Q4 groups were 123.00, 115.00, 113.00, and 92.00 g/L, respectively, with statistically significant differences (*p* < 0.001) (Figure S1b).

**Figure 1.**
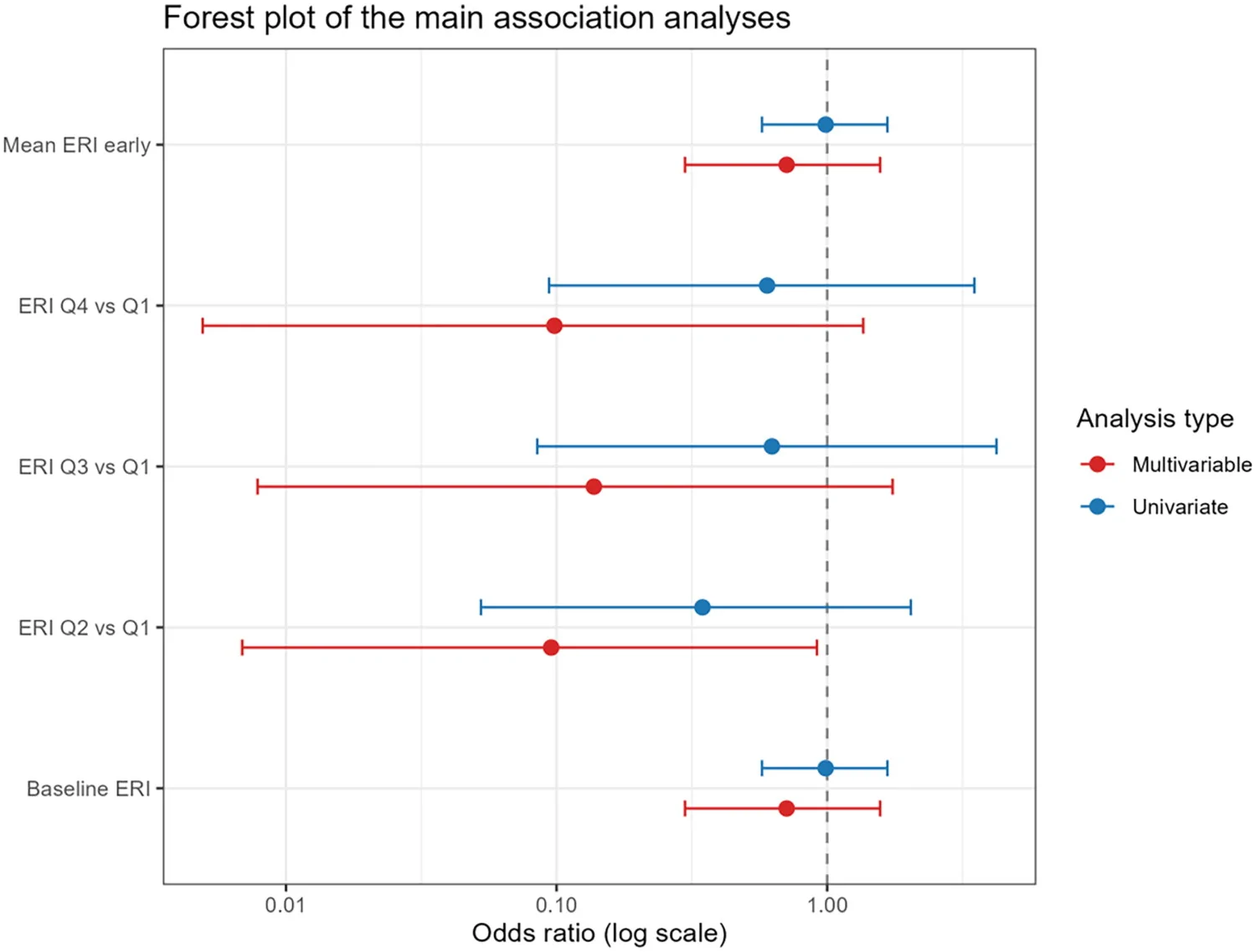
Forest plot of the primary analysis related to baseline ERI.

**Figure 2.**
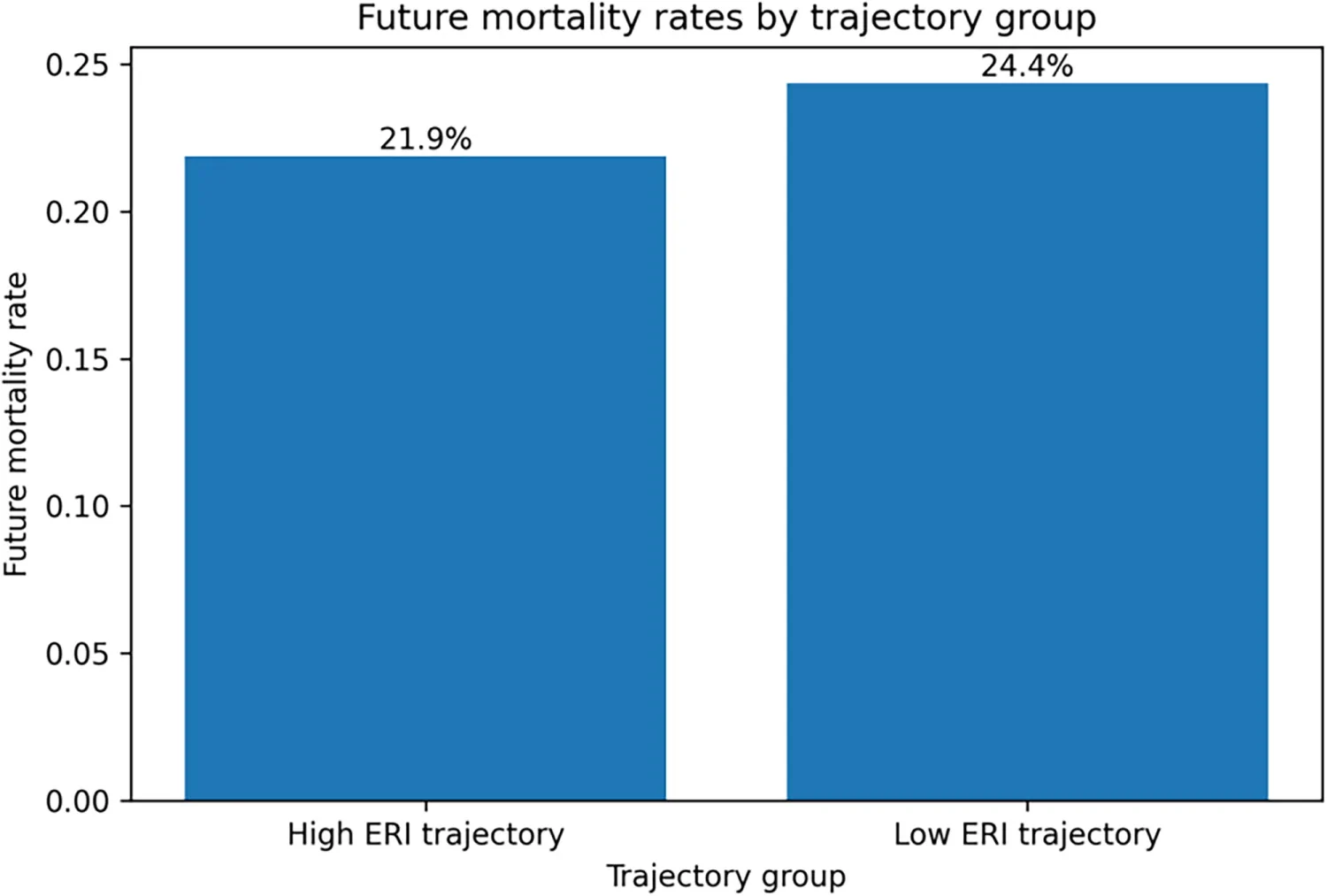
Comparison of ERI trajectory characteristics and future mortality rates.

**Table 1.** Comparison of baseline characteristics by baseline ERI quartile group.

Variable	Q1	Q2	Q3	Q4	p_value
Age (years)	53.00 [46.00, 63.00]	59.00 [48.00, 69.00]	61.50 [44.50, 67.25]	53.00 [42.00, 63.00]	0.167
Dialysis vintage	43.00 [26.00, 50.00]	62.00 [35.00, 84.00]	56.50 [48.25, 62.00]	61.00 [49.00, 76.00]	0.009**
Dry weight (kg)	74.05 [68.50, 81.00]	64.50 [60.00, 72.67]	57.17 [51.92, 66.02]	53.00 [47.50, 60.29]	<0.001***
Hgb (g/L)	123.00 [98.00, 133.00]	115.00 [105.00, 128.00]	113.00 [98.75, 121.00]	92.00 [82.00, 111.00]	<0.001***
Alb (g/L)	36.80 [33.30, 40.70]	36.90 [34.85, 39.45]	37.20 [33.10, 39.00]	36.20 [34.10, 38.10]	0.885
Ferritin (ng/mL)	78.60 [32.30, 220.80]	190.20 [91.90, 334.60]	166.90 [54.80, 274.90]	221.05 [116.37, 389.88]	0.100
CRP	39.50 [29.20, 43.40]	91.70 [37.50, 124.00]	49.70 [31.35, 63.85]	37.80 [17.20, 114.00]	0.848
TSAT (%)	0.00 [0.00, 0.00]	17.16 [17.16, 17.16]	21.23 [21.23, 21.23]	NA	0.368
spKt/V	1.20 [1.20, 1.20]	0.95 [0.88, 1.16]	1.31 [1.30, 1.31]	1.00 [0.97, 1.28]	0.289
URR (%)	65.00 [65.00, 65.00]	56.45 [53.31, 62.11]	66.19 [65.59, 66.78]	58.73 [57.88, 66.45]	0.455
Baseline ERI	2.55 [2.20, 2.79]	3.31 [3.21, 3.40]	3.85 [3.74, 4.00]	4.90 [4.35, 5.26]	<0.001***
Sex: male	28 (75.7%)	30 (81.1%)	23 (63.9%)	19 (51.4%)	0.031*
Sex:female	9 (24.3%)	7 (18.9%)	13 (36.1%)	18 (48.6%)	

Continuous variables are expressed as means ± standard deviation or medians with interquartile range. Categorical variables are expressed as numbers with percentages. *p < 0.05, **p < 0.01, ***p < 0.001.

ERI: erythropoietin resistance index; Alb: albumin; Hgb: hemoglobin; CRP: C-reactive protein; TSAT: predialysis transferrin saturation; spKt/V: single-pool Kt/V; URR: urea reduction ratio.

Furthermore, the gender distribution varied across the different ERI quartiles: the proportion of males was higher in the Q1 and Q2 groups, while the proportion of females was relatively higher in the Q4 group (*p* = 0.031). This variation is also clearly illustrated in the stacked bar chart (Figure S1c). In contrast, differences in age, albumin, ferritin, CRP, TSAT, spKt/V, and URR among the groups were not statistically significant (all *p* > 0.05). Overall, higher baseline ERI levels were primarily associated with longer dialysis vintage, lower dry weight, and lower hemoglobin levels.

### 3.2. Distribution characteristics of future mortality by baseline ERI quartile groups

Further comparison of future mortality rates across different baseline ERI quartile groups revealed some fluctuation in mortality rates among the groups, but no clear increasing trend was observed overall (Figure S2). The bar chart shows that although mortality rates varied across groups, the distribution of mortality risk among different ERI strata did not exhibit a stable dose-response relationship. This suggests that, at the level of rough grouping, baseline ERI may show some association with prognosis; however, this relationship is insufficient to support its use as a stable risk stratification indicator.

### 3.3. Temporal trends in ERI

Quarterly and annual trend analyses revealed that ERI exhibited certain temporal dynamics during follow-up (Figures S3 and S4). The quarterly trend chart shows that the mean and median ERI values fluctuate periodically across quarters, but no sustained unidirectional change is observed overall; the annual trend chart indicates that ERI levels fluctuate only slightly across different years, without forming a clear upward or downward trajectory. This indicates that, in the present study, ERI—as an indicator of low ESA responsiveness—exhibits a certain degree of temporal heterogeneity and fluctuation; however, at the overall level, it does not demonstrate a long-term trend in a single direction.

**Figure 3.**
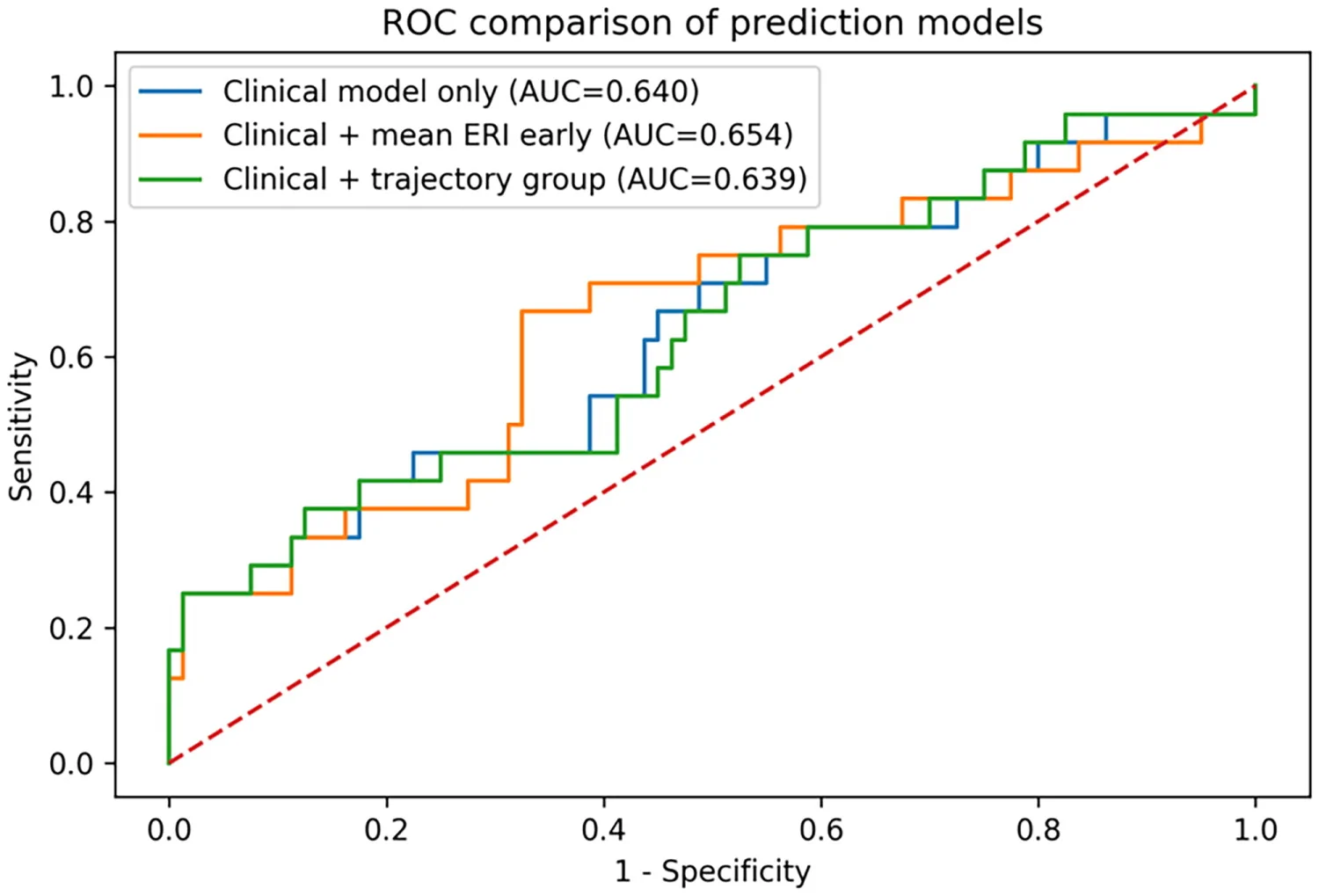
Decision curve analysis diagram of different prediction models.

**Figure 4.**
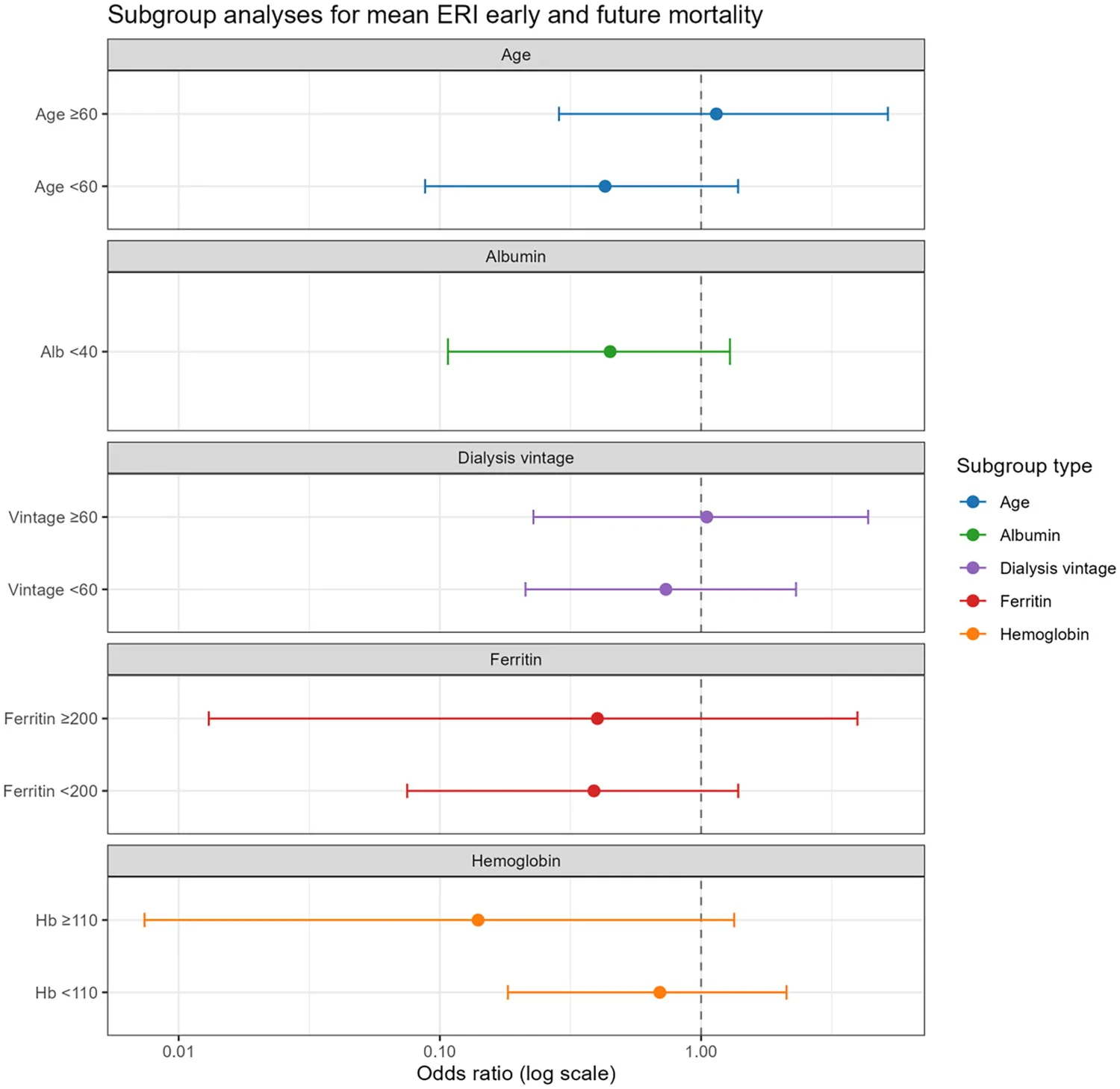
Forest plot of subgroup analysis.

### 3.4. Trajectory characteristics of the two ERI categories based on longitudinal data

To identify long-term patterns of change in ERI, this study compared K-means clustering models with 2, 3, and 4 clusters (Table 2). The results showed that as the number of clusters increased, the within-cluster sum of squares (WCSS) continued to decrease, suggesting an improvement in model fit; however, the silhouette coefficient decreased from 0.274 to 0.252, and the proportion of the smallest category also fell from 45.3% to 12.0%, suggesting that as the number of categories increased, the inter-group separation power and classification stability of the model decreased. Taking into account statistical indicators, category balance, and clinical interpretability, a 2-class model was ultimately selected as the primary analysis model, while the 3-class and 4-class models were used only for exploratory analysis. Overall, ERI exhibited heterogeneous longitudinal variation patterns among MHD patients; although the 4-class model can present more complex variation characteristics, some trajectories cross in the later stages, suggesting that its classification stability and practical application value are limited.

**Table 2.** K-means clustering model.

n_classes	WCSS	silhouette	min_class_pct
2	154.55	0.274	45.3
3	127.72	0.269	22.7
4	105.42	0.252	12

The two-class trajectory model constructed based on longitudinal ERI data classified patients into low-ERI and high-ERI trajectory groups (Figure S5). As shown in the trajectory plots, the two groups remained distinctly separated throughout the follow-up period, with the high-ERI trajectory group exhibiting higher ERI levels than the low-ERI trajectory group in every year, suggesting significant longitudinal heterogeneity in ERI among different patients. This result indicates that, although the overall annual trend is not pronounced, ERI could still differentiate distinct dynamic phenotypes at the individual level.

### 3.5. Association between ERI and mortality outcomes

#### 3.5.1. Logistic regression analysis of baseline ERI and ERI-related indicators with future mortality risk

In univariate logistic regression analysis, regardless of whether early mean ERI or baseline ERI was included in the model as a continuous variable, no significant association was observed between either variable and the risk of future mortality (OR = 0.988, 95% CI: 0.575–1.670, *p* = 0.963) (Table S1, Figure 1). After further adjusting for age, sex, duration of dialysis, albumin, hemoglobin, and ferritin, neither early mean ERI nor baseline ERI (OR = 0.708, 95% CI: 0.298–1.569, *p* = 0.405), still failed to reach statistical significance, suggesting that ERI-related indicators did not demonstrate an independent predictive role for mortality risk in the current sample; when analyzing by baseline ERI quartiles, compared with the Q1 group, the Q2, Q3, and Q4 groups did not show stable and significant differences in mortality risk in either univariate or multivariate models. Although the Q2 group (OR 0.096, 95% CI: 0.007–0.917, *p* = 0.054) showed a marginal trend in the multivariable model, the overall confidence interval was wide, suggesting that the estimate remained unstable. Dialysis vintage (OR 1.032, 95% CI: 1.010–1.060, *p* = 0.010) indicates that longer dialysis vintage is associated with a significantly increased risk of future mortality; hemoglobin (OR 0.947, 95% CI: 0.891–0.997, *p* = 0.055) suggested that patients with higher hemoglobin levels have a relatively lower risk of mortality, consistent with the clinical association between anemia and poor prognosis; however, this finding was not statistically significant. The forest plot further illustrates that while the direction of OR values fluctuates somewhat across different exposure forms, most confidence intervals span 1 (Figure 1).

#### 3.5.2. ERI trajectory characteristics and their association with future mortality risk

However, in the prognostic analysis (Figure 2, Table 3), the future mortality rates for the low ERI trajectory group and the high ERI trajectory group were 24.4% and 21.9%, respectively, with no statistically significant difference (Fisher’s exact test, *p* = 1.000). Univariate logistic regression showed that the future risk of mortality in the high ERI trajectory group compared with that in the low ERI trajectory group (OR 0.733, 95% CI: 0.260–2.070, *p* = 0.558); after adjusting for age, sex, dialysis vintage, albumin, hemoglobin, and ferritin, the adjusted OR was 1.080 (95% CI: 0.341–3.421, *p* = 0.896), suggesting that no significant independent association was observed between the trajectory group and the risk of future mortality.

**Table 3.** Logistic regression analysis of trajectory group models.

Variable	Model	OR_95CI	p
High ERI trajectory (vs Low)	Univariate	0.733 (0.260,2.070)	0.558
High ERI trajectory (vs Low)	Multivariable	1.080 (0.341,3.421)	0.896
Sex (Male vs Female)	Multivariable	1.983 (0.641,6.141)	0.235
Age (years)	Multivariable	1.013 (0.967,1.062)	0.581
Dialysis vintage	Multivariable	0.987 (0.968,1.007)	0.199
Albumin (g/L)	Multivariable	0.912 (0.753,1.104)	0.343
Hemoglobin (g/L)	Multivariable	1.006 (0.964,1.050)	0.788
Ferritin (ng/mL)	Multivariable	0.999 (0.996,1.002)	0.620

ERI: erythropoietin resistance index; OR: odds ratio; CI: confidence interval. *p < 0.05, **p < 0.01, ***p < 0.001.

The results of the trajectory group model (Table 4) showed that the median dialysis vintage values for the low ERI group and the high ERI group were 61.75 (50.00, 82.50) and 81.75 (62.00, 102.69), *p* < 0.05. Dialysis vintage was significantly longer in the high ERI group; the dry weight values were 66.01 (61.87, 70.72) and 54.33 (49.62, 56.85), *p* < 0.001, indicating that the dry weight was significantly lower in the high ERI group. Hemoglobin levels were 119.31 (115.53, 122.86) and 114.12 (110.42, 118.58), *p* < 0.001. Hemoglobin levels were significantly lower in the high ERI group, indicating more severe anemia. The spKt/V values were 1.21 (1.15, 1.31) and 1.33 (1.18, 1.51), *p* < 0.05; the URR values were 65.00 (62.53, 66.40) and 67.06 (63.32, 70.86), *p* < 0.05; dialysis adequacy was higher in the high-ERI group than in the low-ERI group, indicating that the high-ERI group actually had better dialysis clearance, which may be related to lower dry weight and poorer nutritional status in the high-ERI group; ERI-related indicators (Baseline ERI, early mean ERI): both the baseline ERI and early mean ERI were significantly higher in the high-ERI group, *p* < 0.001, consistent with the group definitions. There was a significant difference in gender composition between the two groups, *p* < 0.05. The proportion of females in the high ERI group (50.0%) was significantly higher than that in the low ERI group 26.9%, while the proportion of males in the low ERI group 73.1% was higher. No statistically significant differences were observed in albumin, age, ferritin, or C-reactive protein.

**Table 4.** Trajectory group model comparison.

Variable	Low ERI trajectory	High ERI trajectory	p
Age (years)	57.83 [51.00, 63.50]	57.92 [49.19, 65.31]	0.686
Dialysis vintage	61.75 [50.00, 82.50]	81.75 [62.00, 102.69]	0.004*
Dry weight (kg)	66.01 [61.87, 70.72]	54.33 [49.62, 56.85]	<0.001***
Hgb (g/L)	119.31 [115.53, 122.86]	114.12 [110.42, 118.58]	<0.001***
Alb (g/L)	39.77 [37.96, 41.45]	39.76 [37.79, 41.25]	0.931
Ferritin (ng/mL)	199.35 [111.14, 320.17]	254.77 [164.18, 395.85]	0.209
CRP	69.28 [41.05, 221.72]	55.50 [40.90, 128.00]	0.539
spKt/V	1.21 [1.15, 1.31]	1.33 [1.18, 1.51]	0.048*
URR (%)	65.00 [62.53, 66.40]	67.06 [63.32, 70.86]	0.022*
Baseline ERI	3.52 [3.03, 3.92]	4.76 [4.33, 5.05]	<0.001***
Mean ERI early	3.35 [3.03, 3.54]	4.22 [4.07, 4.52]	<0.001***
Sex: female	21 (26.9%)	16 (50.0%)	0.027*
Sex: male	57 (73.1%)	16 (50.0%)	

Continuous variables are expressed as means ± standard deviation or medians with interquartile range. Categorical variables are expressed as numbers with percentages. *p < 0.05, **p < 0.01, ***p < 0.001.

ERI: erythropoietin resistance index; Alb: albumin; Hgb: hemoglobin; CRP: C-reactive protein; spKt/V: single-pool Kt/V; URR: urea reduction ratio.

Further comparison of the discriminatory ability of different predictive models revealed (Figure 3, Table S2) that the AUC of the clinical baseline model was 0.640; adding early mean ERI increased the AUC to 0.654, while adding the trajectory group resulted in an AUC of 0.639. The results suggest that the trajectory group did not demonstrate superior risk stratification ability compared with a single ERI measurement or early mean ERI. It should be noted that the AUCs reported in this section (0.640–0.654) were derived from models fitted in the trajectory-analysis cohort (*n* = 110) with a restricted set of clinical covariates, whereas the predictive models in Section 3.7 were fitted in the full study cohort (*n* = 150) with a more complete covariate set; the differences in the analysis population and model specification account for the differences in the absolute AUC values.

### 3.6. Nonlinear, Subgroup, and sensitivity analyses

#### 3.6.1. Subgroup analysis and interaction tests

Subgroup analysis results showed that the association between mean early ERI and future mortality risk did not reach statistical significance across different strata of age, albumin, hemoglobin, ferritin, and dialysis vintage (Table S3, Figure 4). Although effect estimates varied somewhat across subgroups, the 95% CI for most groups were relatively wide and spanned 1. Further interaction tests also failed to identify any clear effect-modifying factors, suggesting that no stable subgroup heterogeneity was observed in the current data.

#### 3.6.2. Analysis of nonlinear relationships

Further analysis of the relationship between early mean ERI and future mortality risk using spline models revealed that the curve as a whole did not exhibit a clear and stable nonlinear pattern (Figure 5). Although the OR curve showed some fluctuations in certain intervals, its 95% CI were wide, and no clear threshold effect or dose-response relationship was observed. This suggested that, in the current sample, there was no clear evidence of a nonlinear association between early mean ERI and future mortality.

**Figure 5.**
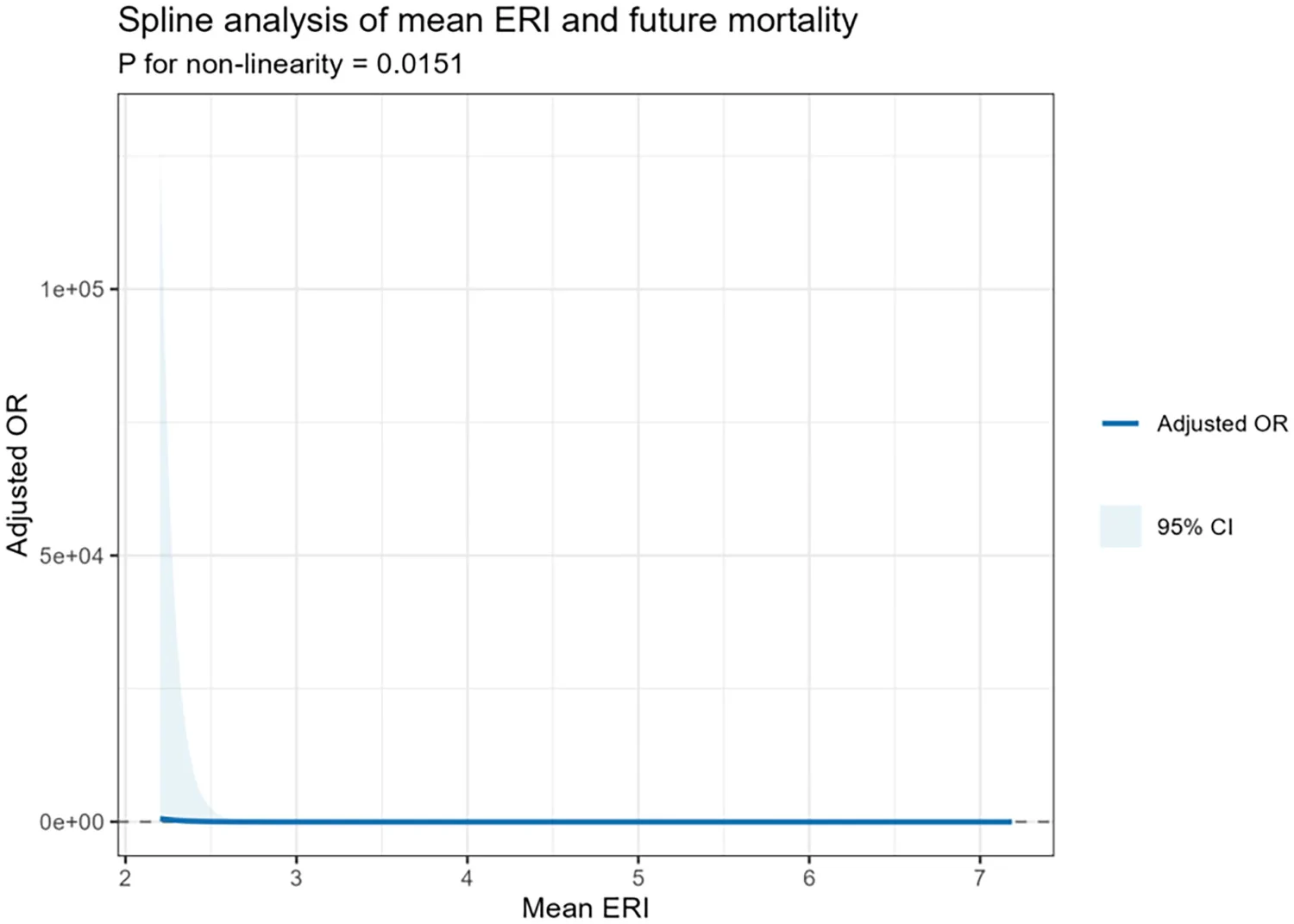
Spline analysis diagram of the relationship between mean ERI early and future mortality risk.

#### 3.6.3. Sensitivity analysis and correlation analysis

In the sensitivity analysis, we log-transformed the early mean ERI and re-ran the model. The results showed that no significant association was observed between log (mean ERI early) and the risk of future mortality (Table 5). The estimated regression coefficient was 0.150, with a 95% CI: 0.003–4.223, *p* = 0.278 (*p* > 0.05), indicating that the conclusions of the main analysis remained consistent overall after changing the form of the variable. There was a significant negative correlation between ERI and hemoglobin (r = −0.337, *p* < 0.001), suggesting that a higher ERI is associated with more severe anemia in patients; there was a significant but weak positive correlation between ERI and ferritin (*r* = 0.227, *p* = 0.044), suggesting that erythropoietin resistance may be associated with iron metabolism; however, there was no significant correlation between ERI and mean ERI early (r = −0.074, *p* = 0.520), or with CRP (r = −0.145, *p* = 0.480), spKt/V (*r* = 0.010, *p* = 0.969), or URR (r = −0.085, *p* = 0.738) (Table 6). These findings indicate that ERI is likely to be a marker for the state of anemia and some aspects of iron metabolism, and has only a modest linear association with dialysis adequacy and inflammatory markers.

**Table 5.** Sensitivity analysis results.

Term	Estimate	conf.low	conf.high	p.value
log_mean_ERI_early	0.150	0.003	4.223	0.278
Age (years)	1.053	0.997	1.122	0.076
Sex: female	5.110	1.184	27.301	0.038
Dialysis vintage	1.028	1.006	1.054	0.016
Alb (g/L)	1.064	0.924	1.233	0.389
Hgb (g/L)	0.946	0.893	0.995	0.043
Ferritin (ng/mL)	0.998	0.994	1.001	0.205

ERI: erythropoietin resistance index; Alb: albumin; Hgb: hemoglobin. *p < 0.05, **p < 0.01, ***p < 0.001.

**Table 6.** Spearman correlation analysis between mean ERI early and relevant clinical indicators.

Variable	r	p_value_fmt
Hgb (g/L)	−0.336973608	<0.001
Alb (g/L)	−0.073946201	0.520
Ferritin (ng/mL)	0.226936629	0.044
CRP	−0.145006848	0.480
spKtV	0.009718718	0.969
URR	−0.084893608	0.738

ERI: erythropoietin resistance index; Hgb: hemoglobin; Alb: albumin; CRP: C-reactive protein; spKt/V: single-pool Kt/V; URR: urea reduction ratio. *p < 0.05, **p < 0.01, ***p < 0.001.

### 3.7. Predictive performance analysis

The AUC of the baseline clinical model was 0.751, and the AUC of the model with continuous ERI values was only slightly higher at 0.753 (Table S4, Figure 6). The DeLong test indicated that the difference between the two models was not statistically significant, suggesting that in the current sample, the inclusion of ERI had a limited effect on improving the model’s discriminatory ability. The AUCs in this section were higher than those reported in Section 3.5 because of the different analysis population and covariate set, as explained above.

**Figure 6.**
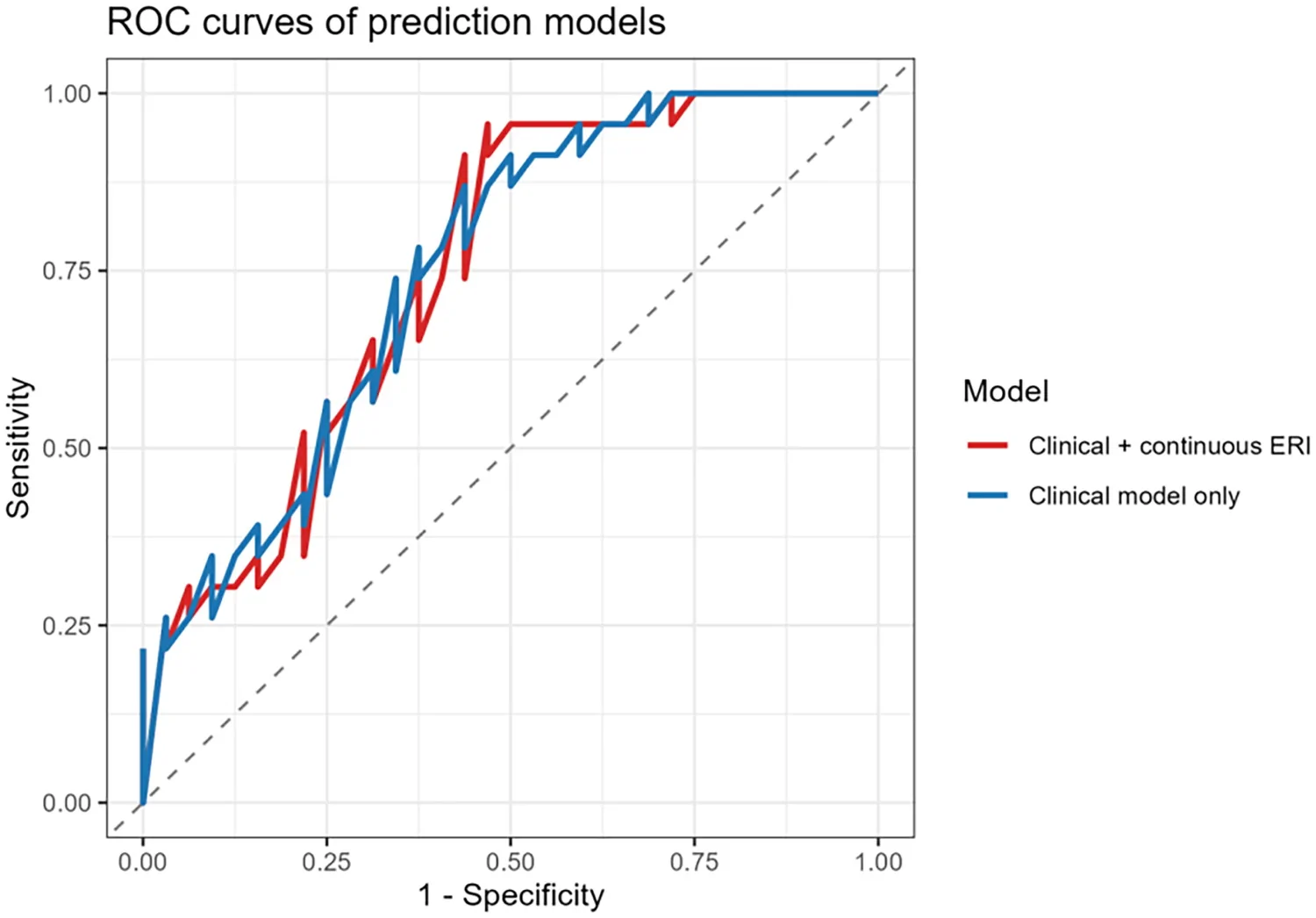
ROC curves of different prediction models.

Decision curve analysis further demonstrated that, across most threshold probability ranges, the difference in net benefit between the baseline clinical model and the model incorporating ERI was minimal, suggesting that ERI provided only limited incremental value for clinical decision support (Figure 7).

**Figure 7.**
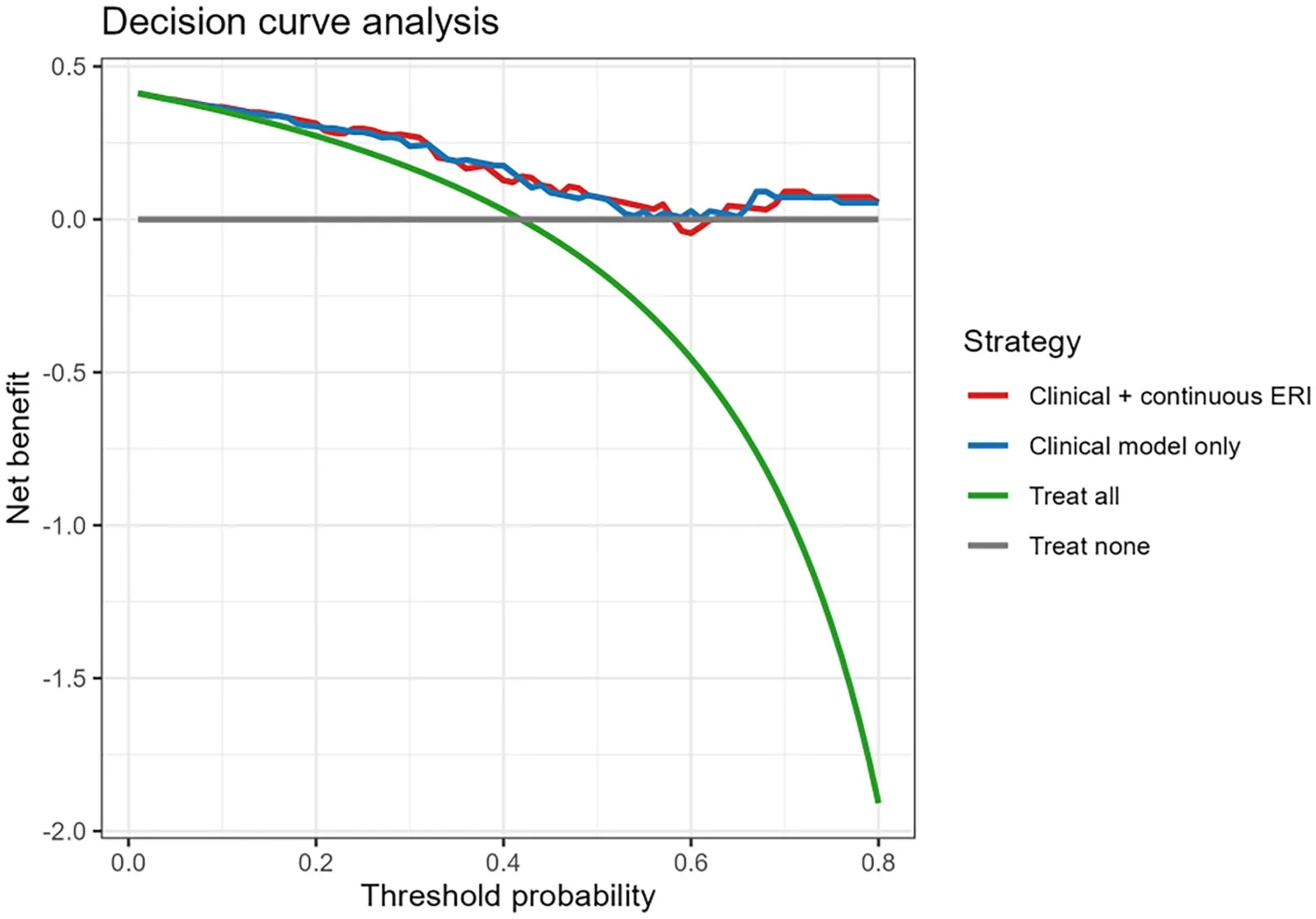
Decision curve analysis diagram of different prediction models.

## 4. Discussion

This study used long-term follow-up data from patients on maintenance hemodialysis (MHD) to identify patterns of change in the erythropoietin resistance index (ERI) using longitudinal data analysis. The study identified that interindividual variability in ERI was significant in patients on maintenance hemodialysis, both in terms of baseline and early-stage mean values, as well as in terms of different longitudinal change trajectories. This finding suggests that ERI is not a static or fixed marker, but rather a clinical phenotype that has dynamic changes.

Further analysis showed that there were significant differences between the patients in the different ERI trajectory groups in terms of dialysis vintage, dry weight, and hemoglobin level, indicating that ERI may to some degree reflect the complexity of patients’ overall clinical status and disease burden. Patients in the higher ERI or high-trajectory groups typically had longer dialysis vintage and lower hemoglobin levels. This finding aligns with the view emphasized in previous studies [9,10] that ‘low ESA responsiveness is dynamic and associated with adverse outcomes.’ Moreover, this study offers a longitudinal depiction of the individual trajectories of ERI development over time, which may be useful for describing patient clinical heterogeneity. This study presents the individual trajectories of ERI change over time from a longitudinal perspective, as opposed to the cross-sectional classification by Cizman et al. [11] who classified low ESA responsiveness as isolated, intermittent, or chronic, offering new research insights into identifying patients who will continue to have high ERI over time.

One of the main results of this study is that, irrespective of the model specification, continuous, categorical or trajectory variable, there was no consistent independent association between ERI and future mortality risk. This suggests that ERI is linked to different clinical conditions; however, the present data do not demonstrate a statistically detectable independent association between ERI and mortality. Thus, ERI should not be regarded as an established prognostic factor on the basis of the present evidence, but rather as a biomarker of a patient’s clinical response that reflects variations in anemia management and underlying pathophysiological conditions. Importantly, the wide confidence intervals of the effect estimates indicate limited statistical precision rather than convincing evidence for the absence of an association. Iron deficiency, chronic inflammation, inadequate dialysis, and secondary hyperparathyroidism are the major factors that contribute to low ESA responsiveness, as summarized by Ogawa and Nitta [12]. In this study, baseline differences existed among the different trajectory groups in terms of dialysis vintage, dry weight, hemoglobin levels, and gender distribution, suggesting that these factors may collectively contribute to the formation and evolution of ERI. Although inflammation and functional iron deficiency are established contributors to ESA hyporesponsiveness, in the present cohort neither ferritin nor CRP differed significantly between the trajectory groups (Table 4), and CRP was numerically lower in the high-ERI trajectory group; these mechanisms, while plausible, were therefore not clearly reflected in our data and warrant direct longitudinal measurement in future studies [13]. Furthermore, the malnutrition-inflammation-atherosclerosis syndrome (MIA) has also been shown to be closely associated with ESA resistance [14]. Therefore, the long-term variability in the clinical course of MHD patients means that ERI is not a static indicator but rather exhibits distinct temporal dynamics. The advantage of trajectory analysis lies in its ability to integrate information on patients’ ESA responses at multiple time points, thereby identifying those who are chronically exposed to high ERI levels; this better reflects the long-term dynamic changes in disease status than a single measurement.

Subgroup analysis revealed no significant effect-modifying roles for age, albumin, hemoglobin, ferritin, or dialysis vintage on the association between ERI and mortality risk, suggesting that this association is generally consistent across different clinical subgroups. There were some differences in the point estimates between some subgroups, such as the relatively larger effect size in the low-Hb group, but none of the interaction tests were statistically significant. Sensitivity analysis results indicated that the overall direction of the estimated effect of ERI on mortality risk was similar across different analytical approaches, including excluding kidney transplant recipients, log-transforming ERI, performing a complete case analysis, and applying different interpolation methods, and that the results did not change significantly. The results of these analyses confirm the robustness of the primary analysis results across various modeling assumptions.

This study has several clinical implications, although they should be interpreted with caution given the largely negative prognostic findings. First, ERI trajectory grouping may serve as a practical descriptive tool for identifying patients with persistently high ESA requirements, and may help clinicians explore potential inflammatory, nutritional, and iron metabolism issues in these patients. In these patients, the standard treatment protocols of slowly increasing the dose of ESA could be ineffective and could even raise the risk of cardiovascular events [9]. Thus, clinical management should focus on the identification and correction of underlying causes, including control of inflammation, optimization of iron therapy, nutritional status, and management of complications such as bone dystrophy, as well as the consideration of novel medications, such as hypoxia-inducible factor prolyl hydroxylase (HIF-PH) inhibitors [15]. These drugs may be more clinically beneficial to patients who do not respond well to standard ESA therapy [16] because they stimulate the production of endogenous erythropoietin and enhance iron utilization. Furthermore, future research could incorporate ERI trajectories with markers of inflammation, iron metabolism, and nutrition to examine their potential role in a comprehensive risk assessment.

This study has some limitations, however. First, no formal sample size or power calculation was performed, and the number of outcome events was limited (26 deaths), which yielded wide confidence intervals and an elevated risk of model overfitting; the study may therefore have been underpowered to detect modest but clinically relevant associations. Second, because exact dates of death were unavailable, logistic regression was used instead of survival analysis; this approach does not account for differences in follow-up duration between patients and may produce biased estimates if observation periods differed substantially, although all patients were observed over the same calendar period. Third, the calculation of ERI is based on ESA dosage and Hgb levels, and the prescribing pattern of ESA, such as how doses are adjusted, how the ESA injections are stored, and how they are administered (intravenous or subcutaneous injection), may vary between dialysis centers, which could influence the standardization of ERI [17]. Fourth, despite multivariable adjustment, there could be some residual confounding factors, such as iron parameters not fully measured, or some inflammatory cytokines. Fifth, this study is an observational study and cannot prove causality; the association of ERI with its trajectory and clinical status may be due to the severity of underlying diseases and treatment burden, not necessarily to a direct causal effect. This finding is in line with the latest KDIGO 2026 update, which does not yet recommend ERI as a routine monitoring indicator for predicting mortality risk in MHD patients [18]. Sixth, the lack of some important data, including inflammatory markers, could have influenced the accuracy of the model, despite the multiple imputation methods used. Last, the potential effect of novel drugs, including HIF-PH inhibitors, on ERI trajectories was not considered in this study, which will be an important area for future research.

Overall, this study shows that ERI in MHD patients is highly longitudinally heterogeneous and that distinct dynamic patterns can be identified. There were some differences in clinical characteristics between ERI levels or trajectory groups, including dialysis vintage, body weight, and hemoglobin level, indicating some phenotypic stratification. However, in the current sample, ERI and its trajectories did not show a statistically detectable independent association with subsequent mortality risk and contributed little to the predictive models; given the retrospective design and limited statistical precision, this should be interpreted as inconclusive evidence regarding the prognostic value of ERI rather than as proof of no association. Thus, ERI is more likely to be a phenotypic marker of pathological states and clinical heterogeneity of anemia than to be a standalone prognostic predictor.

## Supplementary Material

Supplementary_Materials_revised.docx

## Data Availability

The data that support the findings of this study are available from the corresponding author upon reasonable request.
