# Supplementary material for: Longitudinal trajectory characteristics of the erythropoietin resistance index and their association with all-cause mortality in maintenance hemodialysis patients
Source: Ren Fail. 2026 Nov 10;48(1):2756708. doi: 10.1080/0886022X.2026.2756708 (PMC13757841; doi:10.1080/0886022X.2026.2756708)
Supplement: Supplementary file 1 [file IRNF_A_2756708_SM7574.docx]

**Supplementary Materials**

**Table S1 Logistic regression analysis of baseline ERI and ERI-related indicators with future mortality risk**

| model_group | analysis_type | variable | OR_95CI | p_value |
| --- | --- | --- | --- | --- |
| Mean ERI early | Univariate | Mean ERI early | 0.988 (0.575, 1.670) | 0.963 |
| Mean ERI early | Multivariable | Mean ERI early | 0.708 (0.298, 1.569) | 0.405 |
| Mean ERI early | Multivariable | Age | 1.054 (0.998, 1.121) | 0.074 |
| Mean ERI early | Multivariable | Sex | 4.717 (1.117, 24.163) | 0.045 |
| Mean ERI early | Multivariable | Dialysis vintage | 1.027 (1.006, 1.052) | 0.019* |
| Mean ERI early | Multivariable | Albumin | 1.063 (0.924, 1.231) | 0.395 |
| Mean ERI early | Multivariable | Hemoglobin | 0.951 (0.899, 0.999) | 0.058 |
| Mean ERI early | Multivariable | Ferritin | 0.998 (0.995, 1.001) | 0.180 |
| Baseline ERI | Univariate | Baseline ERI | 0.988 (0.575, 1.670) | 0.963 |
| Baseline ERI | Multivariable | Baseline ERI | 0.708 (0.298, 1.569) | 0.405 |
| Baseline ERI | Multivariable | Age | 1.054 (0.998, 1.121) | 0.074 |
| Baseline ERI | Multivariable | Sex | 4.717 (1.117, 24.163) | 0.045 |
| Baseline ERI | Multivariable | Dialysis vintage | 1.027 (1.006, 1.052) | 0.019* |
| Baseline ERI | Multivariable | Albumin | 1.063 (0.924, 1.231) | 0.395 |
| Baseline ERI | Multivariable | Hemoglobin | 0.951 (0.899, 0.999) | 0.058 |
| Baseline ERI | Multivariable | Ferritin | 0.998 (0.995, 1.001) | 0.180 |
| ERI quartiles | Univariate | ERI Q2 vs Q1 | 0.346 (0.053, 2.038) | 0.243 |
| ERI quartiles | Univariate | ERI Q3 vs Q1 | 0.625 (0.085, 4.224) | 0.630 |
| ERI quartiles | Univariate | ERI Q4 vs Q1 | 0.600 (0.094, 3.502) | 0.570 |
| ERI quartiles | Multivariable | ERI Q2 vs Q1 | 0.096 (0.007, 0.917) | 0.054 |
| ERI quartiles | Multivariable | ERI Q3 vs Q1 | 0.137 (0.008, 1.746) | 0.142 |
| ERI quartiles | Multivariable | ERI Q4 vs Q1 | 0.098 (0.005, 1.360) | 0.097 |
| ERI quartiles | Multivariable | Age | 1.066 (1.005, 1.145) | 0.050 |
| ERI quartiles | Multivariable | Sex | 5.236 (1.063, 33.814) | 0.056 |
| ERI quartiles | Multivariable | Dialysis vintage | 1.032 (1.010, 1.060) | 0.010* |
| ERI quartiles | Multivariable | Albumin | 1.036 (0.888, 1.212) | 0.647 |
| ERI quartiles | Multivariable | Hemoglobin | 0.947 (0.891, 0.997) | 0.055 |
| ERI quartiles | Multivariable | Ferritin | 0.998 (0.995, 1.001) | 0.238 |

ERI: erythropoietin resistance index; OR: odds ratio; CI: confidence interval. *p < 0.05, **p < 0.01, ***p < 0.001.

**Table S2: AUC and comparison results of the predictive model**

| model | AUC |
| --- | --- |
| Clinical model only | 0.640 |
| Clinical + mean ERI early | 0.654 |
| Clinical + trajectory group | 0.639 |

AUC: Receiver Operating Characteristic Area Under the Curve

**Table S3: Subgroup analysis and interaction test results of mean ERI early and future mortality risk**

| subgroup | estimate | conf.low | conf.high | p.value | interaction_p |
| --- | --- | --- | --- | --- | --- |
| Age <60 | 0.429 | 0.087 | 1.386 | 0.208 | 0.779656513 |
| Age ≥60 | 1.143 | 0.285 | 5.189 | 0.849 |  |
| Alb <40 | 0.448 | 0.107 | 1.28 | 0.188 | 0.34299549 |
| Hgb <110 | 0.695 | 0.182 | 2.124 | 0.529 | 0.133795027 |
| Hgb ≥110 | 0.139 | 0.007 | 1.338 | 0.119 |  |
| Ferritin <200 | 0.388 | 0.074 | 1.387 | 0.181 | 0.079725627 |
| Ferritin ≥200 | 0.400 | 0.013 | 3.968 | 0.503 |  |
| Dialysis vintage <60 | 0.733 | 0.212 | 2.309 | 0.598 | 0.255810226 |
| Dialysis vintage >=60 | 1.051 | 0.228 | 4.363 | 0.944 |  |

ERI: erythropoietin resistance index; Alb: albumin; Hgb: hemoglobin. *p < 0.05, **p < 0.01, ***p < 0.001.

**Table S4: AUC and comparison results of the predictive model**

| Comparison | AUC |
| --- | --- |
| Clinical model only | 0.751 |
| Clinical + continuous ERI | 0.753 |

AUC: Receiver Operating Characteristic Area Under the Curve

Figure S1 (a) Sample Size Distribution by Baseline ERI Quartile Groups; (b) Boxplot of Key Continuous Variable Comparisons Across Baseline ERI Quartiles; (c) Baseline ERI interquartile gender Broussonetia papyrifera mapping

Figure S2: Comparison of future mortality rates across different baseline ERI quartile groups

Figure S3: Quarterly variation trend of ERI

Figure S4: Annual variation trend of ERI

Figure S5: ERI trajectory diagram based on two types of models
